# Emotional Sensitivity and Work Performance in Lima Firefighters

**DOI:** 10.1002/brb3.70926

**Published:** 2025-10-20

**Authors:** Cholán Castrejón Loida Rocio, Loida Rocio, Salazar Bastos Yamileth Yannira, Yamileth Yannira, Conde Rodríguez Isaac Alex, Isaac Alex

**Affiliations:** ^1^ School of Psychology Universidad Peruana Unión Chosica Peru

**Keywords:** emotional sensitivity, firefighters, sex differences, structural equation modeling, work performance

## Abstract

**Purpose:**

The study aimed to analyze the relationship between emotional sensitivity and work performance in firefighters from Metropolitan Lima, considering potential differences by sex.

**Method:**

A cross‐sectional, predictive, and non‐experimental design was applied with a sample of 366 firefighters. Participants completed the Emotional Sensitivity Scale and the Individual Work Performance Scale. Data analysis included Spearman's correlation and Partial Least Squares Structural Equation Modeling (PLS‐SEM).

**Findings:**

Results revealed a weak but statistically significant negative correlation between emotional sensitivity and work performance (*ρ* = –0.19; *p* = 0.001), with Positive Emotional Sensitivity showing the strongest association (*ρ* = –0.22; *p* < 0.001). Among males, Positive Emotional Sensitivity negatively predicted Work Performance in the Context (*β* = –0.235; *f* ^2^ = 0.050), while Negative Egocentric Sensitivity also showed a negative effect (*β* = –0.190; *f* ^2^ = 0.030). For females, Negative Egocentric Sensitivity exerted a stronger negative influence on Work Performance in the Context (*β* = –0.392; *f* ^2^ = 0.133). No significant associations were found between emotional sensitivity and counterproductive behaviors (*p* > 0.05).

**Conclusion:**

The findings indicate that emotional sensitivity, particularly its positive and negative egocentric forms, may adversely affect work performance in firefighters. These results underscore the importance of developing differentiated emotional regulation and stress management interventions tailored by sex to support occupational performance in high‐demand professions.

## Graphical abstract

This study revealed that emotional sensitivity, particularly its positive and negative egocentric forms, has a negative impact on work performance among firefighters. Using PLS‐SEM, sex differences emerged: Males showed stronger negative links between sensitivity and contextual performance, while females exhibited greater impact on task‐related outcomes. No significant effects were found on counterproductive behaviors. These findings highlight the need for gender‐sensitive interventions in emotional regulation.

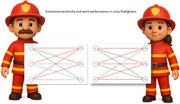

## 1 Introduction

Firefighters are a professional group exposed to extreme conditions of physical and emotional stress due to the nature of their work, which involves attending fires, rescues and medical emergencies, this constant exposure has been associated with a higher prevalence of mental health disorders, such as post‐traumatic stress, depression, anxiety and suicidal ideation, even exceeding the rates observed in the general population (I. H. Stanley et al. 2015; Boffa et al. 2017). These emotional changes have been shown to reduce work performance and undermine operational effectiveness and safety (Lim and Moon 2024).

In this sense, studies carried out by large samples of firefighters have shown that emotional regulation and organizational support play a determining role in mental health and occupational performance. High unregulated emotional labor and negative organizational climates are linked to increased burnout, poorer performance, and higher suicidal ideation in firefighters (Hyun et al. 2020). Likewise, emotional dysregulation and high sensitivity to emotional stimuli have been linked to symptoms of post‐traumatic stress and difficulties in controlling performance‐associated behaviors (Paulus et al. 2018).

Research has shown that anxiety sensitivity and emotional dysregulation are significantly associated with symptoms of post‐traumatic stress, depression, and anxiety among firefighters, which has a direct impact on their work performance (Paltell et al. 2019). Superficial emotional work impairs performance, while deep emotional work shows mixed results; both are influenced by leadership style (Park et al. 2024; Jeung and Chang 2021). Emotional intelligence and proactive coping help prevent emotional exhaustion and improve organizational performance (Wagner and Martin 2012).

Regionally, fewer studies exist, but their results are consistent. Workaholism has been shown to harm volunteer firefighters' performance, primarily through emotional exhaustion, with supervisor recognition lessening this effect (Sandrin et al. 2019). Research indicates that emotional intelligence serves as a moderating factor in the relationship between conflict management style and burnout, underscoring the critical role of emotional competencies in sustaining performance in high‐stress environments (Michinov 2022).

Emotional sensitivity is defined as the individual predisposition to experience intense emotional reactions to both positive and negative stimuli, with associated cognitive, physiological, and behavioral manifestations. It is structured in dimensions such as egocentric negative sensitivity, emotional distancing (ED), and positive interpersonal sensitivity, which reflect different forms of affective reaction and emotional regulation (Guarino and Roger 2005).

Work performance is conceptualized as the set of observable and measurable behaviors that contribute to the fulfillment of organizational objectives, it is divided into three main dimensions: Performance in the task, referring to the effective execution of the assigned functions; contextual performance, which includes voluntary behaviors that favor the organizational environment; and counterproductive behaviors (CB), which negatively affect one's own performance or that of others (Koopmans et al. 2014).

The association between emotional sensitivity and occupational performance has been observed across various settings, with evidence indicating that heightened emotional sensitivity, when coupled with insufficient regulation, may adversely impact both job performance and mental health among firefighters (Paulus et al. 2018; Jeung 2022). Therefore, understanding and defining these variables in a specific way is key to guiding psychological interventions and organizational strategies in fire departments.

In the Latin American context, and specifically in Peru, the connection between emotional sensitivity, emotional regulation, and firefighters' work performance has not been extensively studied, and no published research in indexed journals has been found that directly examines this topic. This situation shows a gap in the scientific literature, considering that working conditions in the region present structural, social, and cultural particularities different from those observed in industrialized countries. Therefore, it is necessary to generate specific empirical evidence that allows validating or contrasting the findings reported in other contexts, contributing to the design of psycho‐emotional intervention programs and strategies aimed at strengthening performance and mental health in the Peruvian emergency corps.

The primary objective of this study is to investigate the relationship between emotional sensitivity and work performance among firefighters in Metropolitan Lima. Specifically, it aims to investigate the relationship between the various dimensions of emotional sensitivity—egocentric negative sensitivity, ED, and positive interpersonal sensitivity—and the components of work performance, encompassing both task fulfillment and contextual and CB. The study also aims to identify possible differences in this relationship depending on sex. Therefore, the study hypothesis was that emotional sensitivity would be negatively associated with work performance in firefighters, with differences according to sex. In addition, the novelty of the present study lies in the use of Partial Least Squares Structural Equation Modeling (PLS‐SEM), which enables the exploration of predictive relationships between emotional sensitivity and work performance, and in the explicit comparison by sex, providing original evidence in the Latin American firefighter population.

## 2 Methodology

### 2.1 Design

The present research has a non‐experimental, predictive, and cross‐sectional design (Coolican 2024).

### 2.2 Participants

The present research was conducted on a sample of firefighters from Central Lima, using a non‐probabilistic convenience sampling method. A total of 366 firefighters took part in the study, all of whom provided their informed consent prior to participation (Conway 2025). The study was conducted between August and September of 2022, in the fire stations of Metropolitan Lima. Inclusion criteria were: Being an active firefighter with at least one year of continuous service and signing informed consent. Exclusion criteria included: Firefighters with less than one year of service, administrative staff not involved in operational activities, retired personnel, and those with incomplete questionnaires. The data that support the findings of this study are available in the online version of the journal (see Supporting Information).

### 2.3 Instruments

*The Work Performance Scale*: It was created by Koopmans (2014) and adapted in Peru by Geraldo Campos (2022). It consists of 19 items with a Likert‐type scale response and divided into three dimensions of individual work performance: Task Performance (6 items), Contextual Performance (8 items), and Counterproductive Work Behavior (5 items). The reliability of this scale is solid, with Cronbach's alpha coefficients greater than 0.80 in each subscale, which indicates good internal consistency and stability of the items in the measurement of individual work performance (Koopmans et al. 2014). In addition, the convergent validity of the scale has been supported by its moderate correlation with other measures of work performance, such as the Health and Work Performance Questionnaire (HPQ) and work engagement scales, which reinforces its usefulness in evaluating different aspects of performance in diverse work contexts (Koopmans et al. 2014). These results suggest that the scale is a reliable and valid resource to evaluate work performance in a comprehensive way in workers from different sectors. (Geraldo Campos 2022). The version applied in this study corresponds to the Spanish adaptation validated in Peruvian workers (Geraldo Campos 2022), which guarantees its cultural and linguistic adequacy.

*The Emotional Sensitivity Scale (ESE)*: This scale measures the emotional responsiveness or reactivity of individuals through 45 items grouped into three dimensions, namely, negative egocentric sensitivity (22 items) (NES), emotional distance (10 items), and positive interpersonal sensitivity (13 items). During its validation, the exploratory factor analysis confirmed a three‐factor structure that reflects the originally proposed dimensions, with a solid internal consistency, reporting Cronbach's alpha coefficients greater than 0.80 in all dimensions (Guarino and Roger 2005). Validation studies also showed that the ESE correlates significantly with related measures, such as health inventories, supporting its convergent validity. NES was associated with physical and psychological symptoms, especially under high‐stress conditions, while positive interpersonal sensitivity predicted empathic responses to social situations (Guarino and Roger 2005). The ESE was used in its Spanish validated version (Guarino and Roger^2005^), ensuring adequate psychometric performance in Hispanic contexts.

### 2.4 Data Analysis

Data processing and analysis were carried out using R software, with the support of the *descr*, *psych*, *PerformanceAnalytics*, *apaTables*, and *seminr* packages, following methodological recommendations for social science analysis (Field et al. 2012). Initially, descriptive statistics were calculated for sociodemographic variables using means, standard deviations, and frequencies. Subsequently, the psychometric properties of the dimensions of emotional sensitivity and work performance were analyzed using measures of central tendency, dispersion, asymmetry, and kurtosis (Revelle 2023). The relationship between the dimensions was explored using Spearman's Rho correlation coefficient, complemented with correlation matrices (Cohen et al. 2014; Peterson and Carl 2020; D. J. Stanley 2021).

For the multivariate analysis, structural equation models were estimated using PLS‐SEM using the seminr package, suitable for exploring relationships between latent variables in relatively small samples. A measurement model composed of the dimensions of the study variables was defined, following the composite creation procedure. The structural model considered relationships from emotional sensitivity variables to work performance variables and was estimated differentiated by sex to evaluate possible structural differences, reporting β coefficients and effect sizes (Hair et al. 2021).

### 2.5 Ethical Aspects

In the present investigation, the following actions were carried out, in the first instance in the same way a letter was presented to the ethics committee of the Peruvian Union University, to obtain the relevant permit, said permit was granted under number 2022‐CE‐FCS‐UPeU‐054, as well as a letter was presented to the administration of the Fire Department. To carry out this research in their respective facilities, the informed consent of each of the people who voluntarily participated in the research was also requested.

## 3 Results

### 3.1 Sociodemographic Data

Table 1 shows the sociodemographic data of the participants, where it is observed that the highest participation was female, with 27.6%. The mean age was 28, with a standard deviation of 7.56. The most frequent origin was the Coast with 76.23%. The most frequent marital status is single, with 63.11%. The mean length of service as an active firefighter is 4.97 years, with a standard deviation of 6.29. The most frequent hierarchical grade is sectional with 58.47%.

**TABLE 1:** Sociodemographic data of the participants.

Variable	M	SD
Age	28.00	7.56
Time of service as an active firefighter	4.97	6.29
Variable	*f*	%
Sex		
Female	101	27.6
Male	265	72.4
Origin		
Coast	279	76.23
Jungle	58	15.85
Mountains	29	7.92
Marital status		
Single	231	63.11
Cohabitant	84	22.95
Married	48	13.11
Divorced	3	0.82
Hierarchical Grade		
Brigadier	7	1.91
Captain	19	5.19
Sectional	214	58.47
Sub‐Lieutenant	78	21.31
Lieutenant	34	9.29
Lieutenant Brigadier	14	3.83

Abbreviations: %, percentage; f, frequency; M, mean; SD, standard deviation.

### 3.2 Normality Analysis

Table 2 shows that the asymmetry and kurtosis scores of the variables are outside the ±2 range, indicating that the distribution of the data is not normal, both for the general sample, the female sample, nor the male sample. Therefore, for the corresponding statistical analyses, non‐parametric tests were used.

**TABLE 2:** Analysis of adjustment to the normal curve of the study variables.

Variables	Mean			Standard deviation			Skewness			Kurtosis		
	G	F	M	G	F	M	G	F	M	G	F	M
**Work performance**	70.58	70.52	70.6	2.32	2.53	2.25	−0.16	−0.15	−0.16	−0.29	−0.34	−0.35
**On task**	26.67	26.54	26.71	1.53	1.51	1.54	−1.52	−1.2	−1.64	5.25	4.9	5.41
**In the context**	30.95	30.78	31.01	1.68	2.01	1.53	−1.06	−1.72	−0.35	3.88	5.44	0.47
**Counterproductive behaviors**	8.46	8.68	8.37	2.15	2.45	2.03	2.78	2.4	2.93	10.4	6.29	12.73
**Emotional sensitivity**	18.93	18.91	18.94	6.17	6.35	6.11	0.22	0.38	0.15	−0.67	−0.57	−0.74
**Negative egocentric sensitivity**	9.39	9.39	9.39	3.21	3.3	3.18	0.09	0.28	0.01	−0.43	−0.43	−0.45
**Emotional distancing**	4.07	4.11	4.06	1.81	1.78	1.83	0.09	0.23	0.04	−0.49	−0.35	−0.56
**Positive emotional sensitivity**	5.47	5.42	5.49	2.33	2.35	2.33	0.23	0.1	0.29	−0.34	−0.46	−0.31

Abbreviations: F, female sample; G, general sample; M, male sample.

### 3.3 Correlation Analysis

Table 3 presents the correlation analysis between emotional sensitivity dimensions and work performance indicators in the general sample. The results show a statistically significant negative correlation between overall Emotional Sensitivity and Work Performance (*ρ* = −0.19, *p* = 0.001), as well as with its specific dimensions: On Task (*ρ* = −0.18, *p* = 0.004) and In the Context (*ρ* = −0.16, *p* = 0.050). Positive Emotional Sensitivity (PES) presents the strongest negative correlation with Work Performance (ρ = −0.22, *p* < 0.001), especially with On Task (*ρ* = −0.17, *p* = 0.011) and In the Context (*ρ* = −0.16, *p* = 0.020). NES also shows a significant negative correlation with Work Performance (*ρ* = −0.15, *p* = 0.007) and On Task performance (*ρ* = −0.15, *p* = 0.037). ED correlates negatively with On Task (*ρ* = −0.15, *p* = 0.006), though not significantly with overall Work Performance. No significant correlations were found between any emotional sensitivity dimensions and CB. All *p*‐values for these correlations are greater than 0.05, indicating non‐significant associations.

**TABLE 3:** Analysis of correlation between the study variables in the general sample.

Variables	Emotional sensitivity		Negative egocentric sensitivity		Emotional distancing		Positive emotional sensitivity	
	*ρ*	*p*	*ρ*	*p*	*ρ*	*p*	*Ρ*	*p*
**Work performance**	−0.19**	0.001	−0.15**	0.007	−0.11	0.073	−0.22***	0.000
**On task**	−0.18**	0.004	−0.15*	0.037	−0.15**	0.006	−0.17*	0.011
**In the context**	−0.16	0.050	−0.14	0.164	−0.11	0.072	−0.16*	0.020
**Counterproductive behaviors**	0.09	0.701	0.08	0.718	0.11	0.223	0.04	0.271

*Note: ρ*, Spearman's Rho correlation coefficient; *p*, significance value.

Table 4 presents the correlation analysis between emotional sensitivity dimensions and work performance indicators, specifically for the female firefighter sample. The results show a statistically significant negative correlation between PES and overall Work Performance (*ρ* = −0.22, *p* = 0.028), suggesting that higher levels of PES may slightly reduce work performance in women. Emotional Sensitivity (total score) also shows a negative correlation with Work Performance (*ρ* = −0.19, *p* = 0.063), though this does not reach conventional levels of statistical significance. For the On Task dimension, Emotional Sensitivity (*ρ* = −0.20, *p* = 0.043) presents a significant negative correlation, indicating that in female firefighters, higher emotional sensitivity tends to be associated with lower task‐specific work performance. The remaining correlations related to On Task and In the Context dimensions are negative but not statistically significant, with *p*‐values above 0.05. No significant correlations were observed between any emotional sensitivity dimensions and CB in the female sample, consistent with the results obtained in the general sample. All corresponding *p*‐values are greater than 0.05, confirming non‐significant associations.

**TABLE 4:** Analysis of correlation between the study variables in the female sample.

Variables	Emotional sensitivity		Negative egocentric sensitivity		Emotional distancing		Positive emotional sensitivity	
	*ρ*	*p*	*ρ*	*p*	*ρ*	*p*	*ρ*	*p*
**Work performance**	−0.19	0.063	−0.17	0.085	−0.06	0.574	−0.22*	0.028
**On task**	−0.20*	0.043	−0.19	0.063	−0.16	0.107	−0.16	0.104
**In the context**	−0.14	0.176	−0.14	0.178	−0.09	0.357	−0.12	0.219
**Counterproductive behaviors**	0.05	0.633	0.05	0.642	0.14	0.170	−0.01	0.895

*Note: ρ*, Spearman's Rho correlation coefficient; *p*, significance value.

Table 5 presents the correlation analysis between emotional sensitivity dimensions and work performance indicators, specifically for the male firefighter sample. The results show that Emotional Sensitivity is significantly and negatively correlated with overall Work Performance (*ρ* = −0.17, *p* = 0.006), suggesting that higher emotional sensitivity is associated with lower work performance in men. This negative relationship is consistent across several dimensions of work performance. Notably, PES shows the strongest negative correlation with Work Performance (*ρ* = −0.19, *p* = 0.002), reinforcing the idea that higher empathy or emotional reactivity may hinder performance in high‐pressure contexts for male firefighters. In the On Task dimension, both Emotional Sensitivity (*ρ* = −0.13, *p* = 0.035) and ED (*ρ* = −0.14, *p* = 0.025) present significant negative correlations, indicating that greater sensitivity and ED are associated with lower task performance. Similarly, PES shows a negative correlation with On Task (*ρ* = −0.13, *p* = 0.039) and In the Context performance (*ρ* = −0.12, *p* = 0.045), highlighting its consistent impact across different performance dimensions in the male sample. No significant correlations were found between emotional sensitivity dimensions and CB. All *p*‐values for these correlations exceed 0.05, indicating non‐significant associations.

**TABLE 5:** Analysis of correlation between the study variables in the male sample.

	Emotional sensitivity		Negative egocentric sensitivity		Emotional distancing		Positive emotional sensitivity	
	*ρ*	*p*	*ρ*	*p*	*ρ*	*p*	*ρ*	*p*
**Work performance**	−0.17**	0.006	−0.13*	0.037	−0.11	0.080	−0.19**	0.002
**On task**	−0.13*	0.035	−0.08	0.204	−0.14*	0.025	−0.13*	0.039
**In the context**	−0.09	0.131	−0.05	0.388	−0.09	0.125	−0.12*	0.045
**Counterproductive behaviors**	−0.05	0.433	−0.04	0.480	0.04	0.557	−0.07	0.244

*Note: ρ*, Spearman's Rho correlation coefficient; *p*, significance value.

### 3.4 Reliability Analysis

Table 6 shows the reliability and convergent validity indicators for both male and female groups using Cronbach's alpha (*α*), composite reliability (ρC), average variance extracted (AVE), and ρA. The results indicate that for both groups, the dimensions WPOT, WPIC, and CB meet or approach the recommended thresholds (α, ρC, ρA ≥ 0.70; AVE ≥ 0.50), suggesting adequate reliability and convergent validity for these constructs. Specifically, CB shows the highest reliability indicators in both groups (*α* = 0.813 for males; *α* = 0.806 for females; AVE = 0.570 and 0.569, respectively), indicating strong internal consistency and variance explained. In contrast, the emotional sensitivity‐related variables (NES, ED, PES) present low reliability and convergent validity indicators in both samples, with *α* values below 0.60, ρC and AVE values particularly low (e.g., ρC = 0.032 for NES in males), suggesting measurement limitations for these constructs. This result highlights a potential need for scale refinement, especially for emotional sensitivity indicators, as they do not reach the commonly accepted psychometric standards (Hair et al. 2021). It is also notable that female participants generally show slightly higher reliability coefficients for emotional sensitivity dimensions compared to males, particularly for NES and PES (*α* = 0.557 and 0.533, respectively, in females, vs. 0.493 and 0.480 in males), which may reflect a more consistent response pattern in the female group regarding these emotional traits. In summary, while the work performance dimensions demonstrate acceptable reliability and convergent validity, the emotional sensitivity measures show weaker psychometric properties, requiring further attention in future studies.

**TABLE 6:** Indicators of reliability and convergent validity: Comparison between male and female groups.

Variable	α Male	α Female	ρC Male	ρC Feale	AVE Male	AVE Female	ρA Male	ρA Female
NES	0.493	0.557	0.032	0.570	0.061	0.105	0.232	0.605
**ED**	0.311	0.288	0.416	0.418	0.118	0.135	0.197	0.297
**PES**	0.480	0.533	0.613	0.630	0.128	0.155	0.426	0.505
**WPOT**	0.597	0.576	0.735	0.734	0.327	0.320	0.608	0.599
**WPIC**	0.618	0.659	0.736	0.758	0.290	0.321	0.602	0.691
**CB**	0.813	0.806	0.868	0.867	0.570	0.569	0.832	0.819

*Note*: Values of α (alpha), ρC (composite reliability), and ρA (construct reliability)≥ 0.70 and AVE (Average Variance Extracted) ≥ 0.50 are recommended for adequate evidence of reliability and convergent validity.

Abbreviations: CB, counterproductive behaviors; ED, emotional distancing; NES, negative egocentric sensitivity; PES, positive emotional sensitivity; WPIC, work performance in the context; WPOT, work performance on task.

### 3.5 Predictive Analysis

Table 7 summarizes the results of the PLS‐SEM model for both male and female firefighter groups, reporting standardized path coefficients (*β*), coefficients of determination (*R* ^2^), and effect sizes (*f* ^2^). NES consistently shows the strongest influence on all dimensions of work performance across both groups. NES presents a more pronounced negative effect on Work Performance on Task (WPOT) in the female group (*β* = −0.351; *f* ^2^ = 0.102; *R* ^2^ = 0.216) compared to the male group (*β* = −0.156; *f* ^2^ = 0.027; *R* ^2^ = 0.109), indicating that higher NES is more closely associated with reduced task performance among women. This pattern repeats in Work Performance in Context (WPIC) and CB, where NES shows higher effect sizes and *R* ^2^ values in females (*f* ^2^ = 0.133 and 0.085, respectively) compared to males (*f* ^2^ = 0.030 and 0.122).

**TABLE 7:** Results of the PLS‐SEM model for male and female groups: β, R^2^ and f^2^.

Regressions	Β Male	Β Female	*f* ^2^ Male	*f* ^2^ Female	*R* ^2^ Male	*R* ^2^ Female
**NES → WPOT**	−0.156	−0.351	0.027	0.102	0.109	0.216
**ED → WPOT**	−0.131	−0.179	0.011	0.030		
**PES → WPOT**	−0.182	−0.021	0.031	−0.000		
**NES → WPIC**	−0.190	−0.392	0.030	0.133	0.131	0.298
**ED → WPIC**	−0.083	−0.150	0.005	0.027		
**PES → WPIC**	−0.235	−0.118	0.050	0.015		
**NES → CB**	0.336	0.343	0.122	0.085	0.144	0.183
**ED → CB**	0.088	0.263	0.006	0.049		
**PES → CB**	0.049	−0.229	0.001	0.037		

Abbreviations: CB, counterproductive behaviors; ED, emotional distancing; *f* ^2^, size of Cohen's effect; NES, negative egocentric sensitivity; PES: positive emotional sensitivity; *R* ^2^, coefficient of determination; WPIC, work performance in the context; WPOT, work performance on task; β, standardized path coefficient.

ED has weaker path coefficients overall, but slightly higher values in the female group, especially with CB (*β* = 0.263; *f* ^2^ = 0.049), compared to males (*β* = 0.088; *f* ^2^ = 0.006). This suggests that ED contributes more noticeably to CB in women.

PES shows smaller and more variable effects. In the male group, PES is negatively associated with WPOT and WPIC (*β* = −0.182 and *β* = −0.235, respectively), while in the female group, these effects are weaker or even opposite in direction, particularly concerning CB (*β* = −0.229; *f* ^2^ = 0.037), suggesting that higher PES is linked to fewer CB among female firefighters.

The coefficients of determination (*R* ^2^) are consistently higher in the female group, indicating that the model explains a greater proportion of variance in work performance outcomes among women (WPOT *R* ^2^ = 0.216; WPIC *R* ^2^ = 0.298; CB *R* ^2^ = 0.183) than among men (WPOT *R* ^2^ = 0.109; WPIC *R* ^2^ = 0.131; CB *R* ^2^ = 0.144). This reinforces the importance of considering sex differences when analyzing how emotional sensitivity influences work performance.

**FIGURE 1:**
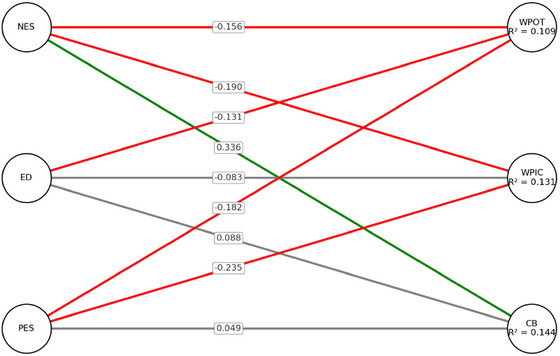
Path diagram of male model.

Figure 1 displays the path diagram for the male model, illustrating the relationships between the emotional sensitivity dimensions (NES, ED, PES) and work performance outcomes (WPOT, WPIC, CB).

**FIGURE 2:**
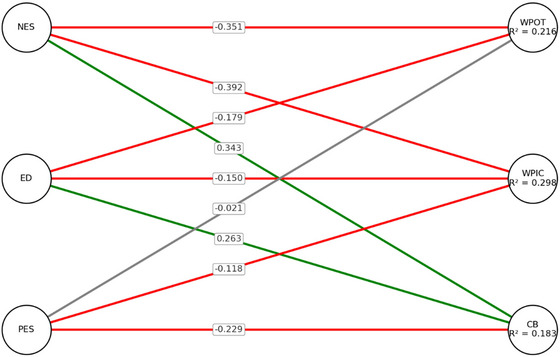
Path diagram of female model.

Figure 2 displays the path diagram for the female model, illustrating the relationships between the emotional sensitivity dimensions (NES, ED, PES) and work performance outcomes (WPOT, WPIC, CB).

## 4 Discussion

This study contributes novel evidence by applying PLS‐SEM modeling and differentiating the analysis by sex, methodological strategies scarcely used in research with firefighters in Latin America. These innovations strengthen the robustness of the results and highlight the added value of the present investigation. The objective of this study was to analyze the relationship between emotional sensitivity and work performance in firefighters from Metropolitan Lima, specifically exploring differences by sex through both correlation analyses and predictive modeling using PLS‐SEM.

In the general sample, a weak but statistically significant negative correlation was observed between emotional sensitivity and work performance (*ρ* = −0.19, *p* = 0.001), particularly for PES (*ρ* = −0.22, *p* < 0.001). This result is consistent with studies that identified negative effects of surface acting on job performance among firefighters (*β* = −0.315) (Park et al. 2024), positive associations between emotional labor and burnout across five sub‐dimensions (*b* = 0.111–0.149) (Jeung 2022), and reductions in job burnout related to mindfulness and reappraisal strategies (Jin et al. 2024). Theoretically, these findings are consistent with the Job Demands–Resources (JD‐R) model, which states that high job demands, such as emotional labor, require matching personal and organizational resources to avoid negative outcomes like reduced performance (Bakker and Demerouti 2017; Michinov 2022).

Among male firefighters, stronger and more consistent negative relationships were identified. PES correlated negatively with work performance (*ρ* = −0.19, *p* = 0.002), with PLS‐SEM path coefficients such as PES → WPIC (*β* = −0.235, *f* ^2^ = 0.050) and NES → WPIC (*β* = −0.190, *f* ^2^ = 0.030). These findings align with evidence of significant associations between job stress, burnout (*r* = 0.719), and reduced cognitive performance (*r* = −0.512 to −0.757) (Mehrifar et al. 2025), as well as differences in cognitive performance and emotion regulation related to PTSD (*p* < 0.01) (Shavaran and Tavakoli 2020), and emotional intelligence moderating burnout outcomes (*p* < 0.01) (Michinov 2022). From a theoretical standpoint, these results support the conservation of resources (COR) theory, which posits that individuals strive to retain, protect, and build resources and that stress occurs when resources are threatened or lost—particularly relevant in men with lower emotional coping capacity (Hobfoll 1989; Mehrifar et al. 2025).

For female firefighters, although the relationships were generally weaker, PES maintained a significant negative correlation with work performance (*ρ* = −0.22, *p* = 0.028). In the PLS‐SEM model, NES → WPIC exhibited a higher path coefficient compared to the male group (*β* = −0.392, *f* ^2^ = 0.133). This pattern is in line with studies documenting emotional labor challenges related to organizational discrimination among female firefighters (Choi et al. 2023), differences in emotional regulation and psychological well‐being by sex (*r* = 0.488, *p* < 0.001) (Toaza Caisaguano and Pilco Guadalupe 2025), and improvements in psychological well‐being and self‐efficacy through emotion regulation training (Sharifi et al. 2021). Theoretically, this is consistent with gender role strain theory, which suggests that gendered expectations shape emotional regulation styles and outcomes, with women generally socialized to exhibit better emotional control but still susceptible to overload in extreme contexts (Levant and Alto 2017).

Across both male and female groups, no significant correlations were observed between emotional sensitivity and CB (all *p* > 0.05). This observation is consistent with research reporting that while emotional labor is related to turnover intention (*β* = 0.29), it is moderated by organizational support rather than directly influencing negative behaviors (Lim and Moon 2023), effects of guanxi and emotion regulation strategies in burnout linked to work–family conflict (Wu et al. 2019), and conscientiousness and cognitive reappraisal reducing anxiety and depression symptoms in firefighters (Tao et al. 2022). This pattern is theoretically aligned with social exchange theory, which emphasizes that perceptions of fairness, organizational support, and reciprocity govern workplace behaviors more strongly than individual emotional characteristics (Ahmad et al. 2022).

This study presents limitations that must be considered when interpreting the findings. First, the cross‐sectional and correlational design prevents establishing causal relationships between emotional sensitivity and work performance. Although PLS‐SEM modeling provided predictive insights, longitudinal designs would be necessary to confirm temporal precedence and causality. Second, the use of non‐probabilistic convenience sampling limits the generalizability of the results. The sample was predominantly male and limited to firefighters from Central Lima, introducing gender imbalance and geographical bias. It is recommended that future studies increase female representation and include firefighters from other Peruvian regions to enhance external validity. Third, the emotional sensitivity scales used showed low reliability and convergent validity indicators, particularly for NES and ED (*α* < 0.70; AVE < 0.50), suggesting potential issues in scale adaptation or cultural relevance. Future research should prioritize reviewing and validating these instruments through advanced psychometric methods such as CFA or IRT. Other relevant variables, such as organizational climate, perceived stress, social support, and leadership styles, were not included in the model. These factors have been identified as important moderators and mediators in the relationship between emotional regulation and job performance in emergency services. In addition, the characterization did not include relevant biomedical or behavioral variables such as weight, body mass index (BMI), waist circumference, levels of physical activity, or history of chronic diseases, which restricts the possibility of comprehensively analyzing factors associated with work performance.

The findings of this study have several important practical implications for the management, training, and psychological support of firefighters in Lima and similar contexts. The identification of a weak but consistent negative relationship between emotional sensitivity, particularly PES and NES, and work performance suggests the need to develop targeted emotional regulation and stress management programs within firefighter institutions. These programs should prioritize equipping firefighters with cognitive‐behavioral strategies and emotion regulation techniques aimed at reducing the negative impact of excessive emotional reactivity on operational efficiency. Given that male firefighters showed stronger negative relationships, interventions should specifically address gender differences, providing differentiated training modules and support mechanisms tailored to the emotional regulation profiles of male and female firefighters.

Furthermore, the results reinforce the importance of integrating mental health programs as part of the professional development and occupational safety frameworks in fire departments. Institutional policies should include routine psychological evaluations, resilience training, and access to counseling services to mitigate the potential impact of emotional sensitivity on job performance. The absence of a significant relationship between emotional sensitivity and CB indicates that work performance is mainly influenced by emotional demands rather than by negative intentional actions, which highlights the value of fostering supportive organizational climates and promoting fair leadership practices. By incorporating these considerations into institutional policies, firefighter organizations could improve both the mental health and operational effectiveness of their personnel, ensuring better service delivery to the community under emergency conditions.

In conclusion, this study identified a weak but consistent negative relationship between emotional sensitivity and work performance in firefighters from Metropolitan Lima, with stronger effects observed among male personnel. PES and Negative Egocentric Sensitivity were the dimensions most closely associated with lower performance, particularly in task‐related and contextual activities. Although these effects were not large, they underscore the relevance of emotional regulation as a factor influencing operational efficiency. The absence of significant associations with CB suggests that emotional sensitivity primarily affects cognitive and procedural aspects of performance rather than intentional negative actions. These findings highlight the importance of implementing gender‐sensitive emotional support and stress management programs within firefighter institutions to promote both mental health and optimal work performance.

## Author Contributions

**Cholán Castrejón Loida Rocio** conceptualization, investigation, writing – original draft, methodology, validation, software, formal analysis, project administration, resources. **Salazar Bastos Yamileth Yannira** conceptualization, investigation, writing – original draft, methodology, validation, software, formal analysis, project administration, resources. **Conde Rodríguez Isaac Alex** conceptualization, investigation, writing – original draft, methodology, validation, software, formal analysis, project administration, resources, visualization, writing – review & editing, data curation, supervision.

## Conflicts of Interest

The authors declare that they have no conflicts of interest regarding the publication of this manuscript.

## Peer Review

The peer review history for this article is available at https://publons.com/publon/10.1002/brb3.70926.

## Supporting information



Supplementary Information

## Data Availability

The data that support the findings of this study are available in the Supplementary Material section.
